# Magneto-Plasmonic Nanoparticle Grid Biosensor with Enhanced Raman Scattering and Electrochemical Transduction for the Development of Nanocarriers for Targeted Delivery of Protected Anticancer Drugs

**DOI:** 10.3390/nano11051326

**Published:** 2021-05-18

**Authors:** Hoda Ilkhani, Chuan-Jian Zhong, Maria Hepel

**Affiliations:** 1 Department of Chemistry, State University of New York at Potsdam, Potsdam, NY 13676, USA; 2 Central New Mexico Community College, Albuquerque, NM 87106, USA; 3 Department of Chemistry, Binghamton University, Binghamton, NY 13902, USA; cjzhong@binghamton.edu

**Keywords:** Raman biosensor, Au-coated nanoparticles, hot-spot SERS substrate, anticancer drug nanocarriers, targeted drug delivery sensing, magneto-plasmonic nanoparticles

## Abstract

Safe administration of highly cytotoxic chemotherapeutic drugs is a challenging problem in cancer treatment due to the adverse side effects and collateral damage to non-tumorigenic cells. To mitigate these problems, promising new approaches, based on the paradigm of controlled targeted drug delivery (TDD), and utilizing drug nanocarriers with biorecognition ability to selectively target neoplastic cells, are being considered in cancer therapy. Herein, we report on the design and testing of a nanoparticle-grid based biosensing platform to aid in the development of new targeted drug nanocarriers. The proposed sensor grid consists of superparamagnetic gold-coated core–shell Fe_2_Ni@Au nanoparticles, further functionalized with folic acid targeting ligand, model thiolated chemotherapeutic drug doxorubicin (DOX), and a biocompatibility agent, 3,6-dioxa-octanethiol (DOOT). The employed dual transduction method based on electrochemical and enhanced Raman scattering detection has enabled efficient monitoring of the drug loading onto the nanocarriers, attaching to the sensor surface, as well as the drug release under simulated intracellular conditions. The grid’s nanoparticles serve here as the model nanocarriers for new TDD systems under design and optimization. The superparamagnetic properties of the Fe_2_Ni@Au NPs aid in nanoparticles’ handling and constructing a dense sensor grid with high plasmonic enhancement of the Raman signals due to the minimal interparticle distance.

## 1. Introduction

With the new advances in nanobiotechnology, the targeted drug delivery (TDD) of cytotoxic chemotherapeutics is becoming one of the most promising approaches in cancer treatment [1,2,3]. The TDD systems are based on drug nanocarriers, equipped with biorecognition capability, directed toward cancer cells [4,5]. Further interests in TDDs arise from their ability to mitigate both the adverse side effects of drugs and collateral damage to non-tumorigenic cells, which cannot easily be achieved with the systemic drug administration used in classical chemotherapy. Moreover, there are indications that a TDD may protect the drugs against inactivation by the biological medium, and likely alleviate the multidrug resistance of tumors [6]. In this study, we have investigated new platforms for designing and testing model drug nanocarriers for future applications in new TDD systems. The proposed novel biosensing platform is based on a monolayer of model nanocarriers immobilized on a Au(111) substrate or a gold disk electrode (AuDE). The nanocarriers consist of superparamagnetic gold-coated Fe_2_Ni@Au core–shell nanoparticles, functionalized with a chemotherapeutic drug doxorubicin (DOX) and other functional molecules. For the sensitive monitoring of analytical signals associated with drug loading and releasing, a dual surface-enhanced Raman light scattering (SERS) and electrochemical transduction has been employed. The proposed biosensing platform may serve as the developmental tool for basic investigations involving the design and testing of new nanodrugs and for studies of controlled drug release from nanocarriers under simulated intracellular conditions.

Chemotherapy has widely been recognized as one of the more successful methods in cancer therapy. Classical chemotherapy, based on a systemic administration of highly cytotoxic drugs, is able to preferentially kill cancer cells. Unfortunately, this therapy has serious disadvantages, such as the severe side effects, high collateral damage to healthy cells, and it may lead, in some cases, to organ failure or systemic collapse. Therefore, novel approaches have been aimed at replacing classical chemotherapy with nanotechnology-driven theranostic methods that can mitigate its drawbacks. The new approaches are based on the targeted delivery of anticancer drugs directly to cancer cells using specially designed drug nanocarriers.

A variety of nanoparticles (NPs) have been designed and investigated for applications as the nanocarriers in TDD, including liposomes [7], solid lipid NPs [8], empty-shell biomolecules, such as apoferritin [9] or cyclodextrin [10], biodegradable particles [11], polymeric dendrimer nanostructures [4], plasmonic [12,13] and magnetic [14] NPs, and others. 

The immobilization of drugs, targeting ligands, and other functional molecules onto the nanoparticle nanocarriers involves various kinds of binding, including electrostatic interactions [15,16], covalent binding [12,13,14,17,18], adsorption [19], supramolecular interactions [9,20], and trapping [7]. The drug binding to nanocarriers and encapsulation processes have been carried out to improve pharmacological and therapeutic effects of the drugs in terms of reducing collateral damage and side effects [21,22,23], while increasing the local drug potency at the targeted cancer cells. The modality of drug binding to nanocarriers has to be carefully assessed, also taking into account the specificity of the subsequent controlled drug release processes at the target tumor cells [24,25,26].

The drug-loaded nanocarriers themselves require protection against damage due to the immune system response and opsonization [27,28,29]. In order to attain proper biocompatibility, a nanocarrier is ordinarily functionalized by the immobilization of pegylated ligands on its surface [28,30]. Biocompatible polyelectrolyte complex nanostructures and NPs with other coatings can also be designed to serve as the TDD nanocarriers [29,31]. On the other hand, biogenic nanocarriers, such as those based on apoferritin nanocage carriers [9], generally do not require any extra biocompatibility ligands.

Doxorubicin, used in this work as the model chemotherapeutic drug, is one of the most widely used anticancer drugs [32]. It is especially effective in the treatment of breast cancer, lymphoma, leukemia, bladder cancer, Kaposi’s sarcoma, and others. Upon entry into a cell, DOX molecules travel to the nucleus, intercalate into the DNA duplex, and act against topoisomerase II, an enzyme active in DNA replication and repair. The DOX intercalation results in DNA cleavage [33]. There are several disadvantages of cancer treatment with DOX, including high toxicity to healthy cells, serious side effects, low retention of the drug, and low solubility in aqueous solutions. The side effects include hair loss, vomiting, rash, and mouth inflammation. Additionally, cardiotoxicity and myelosuppression, due to DOX, are the results of local toxicity, occurring at the regular drug doses [34]. In our recent study, to mitigate the adverse side effects of a chemotherapeutic drug gemcitabine, we have applied binding of the drug molecule to a nanocarrier via its most active functional group [13]. Such binding deactivates the drug while it is transported on the nanocarrier through a biological environment. Therefore, in this work, DOX was bound to a linker, mercaptopropionic acid (MPA), immobilized on gold nanoparticles (GNPs) or magnetic nanoparticles (MNPs) by its –NH_2_ functional group. It has been demonstrated that this kind of approach enables increasing the local drug dosing at the target cancer cells, thus improving the treatment efficacy [35].

Folic acid receptors (FRs), found to be overexpressed in different types of cancer cells including breast cancer [4], serve as the main targeted receptors, specific to cancer cells. Normal cells, in contrast, express only a very small number of FRs, localizing them on the apical surface of polarized epithelia, where the receptors remain inaccessible to the drugs [36]. Hence, nanocarriers with folate conjugates can selectively interact with cancer cells, by recognizing the overexpressed FRs, and be internalized in cancer cells via the receptor-mediated endocytosis. By this mechanism, folic acid-covered and drug-loaded nanocarriers can overcome drug resistance caused by P-glycoprotein efflux pumps [37]. Thus, the availability of folic acid receptors in cancer cell membranes makes them very efficient agents for drug targeting [38].

For the detection of subtle changes in surface concentrations of active compounds, surface-enhanced Raman spectroscopy (SERS) is often utilized due its high sensitivity and functional group recognition capability [39,40]. In previous studies, we have employed SERS for the analysis of DNA damage caused by strong oxidants and drugs [17], and for the monitoring of chemotherapeutic drug upload and release from nanoparticle nanocarriers [12,13,41,42,43]. Raman spectroscopy and plasmonic effects were recently employed by the Brolo group for the detection of radiation responses in non-small cell lung cancer [44] and in biosensors for breast cancer antigens [45], as well as for the development of immunoassays for the detection of Zika virus [46]. The quantitative Raman assays have also been developed for various solid drug dosing formats, such as capsules, tablets, and powders [47,48,49]. In addition, Gotter et al. [50] have described the use of Raman spectroscopy for the quantification of a drug suspended in a simple semi-solid formulation consisting of paraffin. More complex solid pharmaceutical formulations were analyzed by Hargreaves et al. [51], who established Raman spectroscopy for the quantitative analysis of multi-component pharmaceutical capsules. Furthermore, Stillhart et al. [52] have demonstrated a method suitable for the quantification of low-level excipients in the formulation. Raman spectroscopy is very useful in drug analysis, not only due to the high sensitivity and chemical specificity, but also due to the ease of use, minimal sample handling, and significant discrimination against packaging materials and tablet excipients [53]. The addition of electrochemical sensing to SERS monitoring enables convenient characterization of the properties of nanocarriers, because voltammetric techniques offer high sensitivity and scalability and can be readily integrated in various types of biosensors [12,17]. Recently, a variety of SERS substrates with high signal amplifications due to overlapping plasmonic fields have been proposed for biosensor applications, including localized surface plasmon (LSP) coupling demonstrated by the appearance of enhancement “hot spots” for Raman scattering [12,17,39,40,41,54], as well as the coupling of multiple plasmon modes [55,56], including LSPs, surface plasmon polaritons (SPPs), and bulk plasmon polaritons (BPPs). For very dilute analytes and trace concentrations, various kinds of molecular enrichment, including capillary action with solvent evaporation, magnetic separation, molecular imprinting, and others, have recently been applied [57,58].

By using magnetic nanoparticle-based drug nanocarriers and external magnetic field focusing, it has become possible to directly move and localize drugs to solid tumors to reduce the side effects of anticancer drugs [34,42,59,60]. Magnetic nanoparticles offer the benefit of utilizing both the enhanced permeability and retention (EPR) effect (referred to as the passive targeting) whilst also ensuring a direct, guided delivery to the tumor (active targeting) [14,59,61,62]. Another advantage of this nanotechnology is the enhancement of magnetic resonance imaging and induction of cytotoxicity through near-infrared derived hyperthermia [60,63]. Unfortunately, many magnetic nanoparticles, such as the superparamagnetic iron oxide, Fe_3_O_4_ (magnetite), are unstable without protection, and in physiological media undergo degradation due to oxidation, aggregation, and precipitation. Additionally, the functionalization of non-metallic magnetic NPs is challenging [62,64]. In vivo, this may result in the early release of anticancer drugs whilst still in the bloodstream, and failure of the drug to reach the tumor [36]. Therefore, to address these problems, we and others have developed new magnetic-alloy carriers, such as the superparamagnetic Fe_2_Ni@Au core–shell nanocarriers [14,17,42,65]. The Au shell provides a convenient platform for surface functionalization of the NPs. Different kinds of macromolecules, such as peptides [37] and polymers [38,66], or hydrophilic ligands forming a self-assembled monolayer (SAM) [64,67], can be utilized for this purpose. 

Herein, we report on the development of a new magneto-plasmonic grid sensor with electrochemical and surface-enhanced Raman scattering (SERS) transduction, enabling the investigation of drug nanocarriers, including functionalization with targeting ligands, attachment of biocompatibility enhancing molecules, and monitoring of drug uploading and release. The SERS sensor is based on a Au(111) substrate with a monolayer of gold-coated core–shell magneto-plasmonic nanocarriers, Fe_2_Ni@Au, with inter-particle plasmonic field “hot-spot” enhancement. The utility of the proposed methodology was demonstrated in the case of the development of a model drug nanocarrier for the targeted delivery of an anticancer drug, doxorubicin (DOX). The model nanocarrier consists of a superparamagnetic Fe_2_Ni@Au NP, which is further functionalized with DOX, folic acid targeting ligand, and 3,6-dioxa-octanethiol biocompatibility agent. All steps of the nanocarriers synthesis and testing are described in detail. The proposed biosensing device and the methodology developed can be utilized in designing and testing other nanocarriers for TDD systems. Due to the expansive increase in cancer cases, many nanocarriers of different chemical compositions must be urgently investigated to find the best solutions for treatment of this lethal disease. In this study, we selected Fe_2_Ni@Au magnetic nanoparticles because they are more stable than commonly used Fe_3_O_4_ nanoparticles [14], show excellent superparamagnetic and plasmonic properties, and can be readily modified by chemotherapeutic drugs, targeting ligands, and biocompatibility agents due to the simple conjugation mechanisms, to serve as the drug nanocarriers.

## 2. Materials and Methods

### 2.1. Materials

The anticancer drug doxorubicin (DOX) used in this work was received from Selleck Chemicals Inc. (Houston, TX, USA). Chemical compounds used for the functionalization of nanocarriers and linking, including 1-octanethiol (OT), 1,6-hexanedithiol (HDT), 3,6-dioxa-octanethiol (DOOT), 3,6-dioxa-1,8-octanedithiol (DOODT), folic acid (FA), cysteamine hydrochloride (CYS), 5-phenyl-1,3,4-oxadiazole-2-thiol (PODAT), and 4-aminothiophenol (PATP), were acquired from Sigma-Aldrich Corp. (Milwaukee, WI, USA). Covalent binding of functional ligands was achieved using N-(3-dimethylaminopropyl)-N’-ethylcarbodiimide hydrochloride (EDC) and N-hydroxysuccinimide (NHS) (Sigma-Aldrich Corp.) to form an amide bond. All reagents were of analytical grade and were used as received. The reduced graphene oxide nanosheets (rGO) were obtained by oxidation of graphite flakes in acidic permanganate solution at 50 °C, followed by partial electrochemical reduction, using a method we have recently developed for the synthesis of electrocatalysts for polyphenol sensors [68]. The Au(111) gold substrates (250 nm thick, deposited on a borosilicate glass coated with a 4 nm Cr adhesion layer) were obtained from Arrandee, GmbH (Werther, Germany). The magnetic nanoparticles, Fe_2_Ni, for Fe_2_Ni@Au nanocarriers were synthesized and tested according to the procedure published elsewhere [65], with some modifications. All chemicals were obtained from Sigma-Aldrich Corp. (Milwaukee, WI, USA) Briefly, FeSO_4_·7H_2_O and NiCl_2_ were dissolved in 100 mL of deionized water and pH-adjusted to 5.8 with NaOH. Then, ethylene glycol was used as the surfactant and 4 mL of hydrazine hydrate was used as the reducing agent. The mixture was reacted in an autoclave at 120 °C for 19 h under nitrogen atmosphere. In the present study, the hydrothermal system with an appropriate feeding ratio enabled obtaining monodispersed spinel-type Fe_2_Ni NPs with superparamagnetic properties and high resistance against oxidation. The Fe_2_Ni NPs were coated with a layer of Au, typically 10 nm thick, by modifying the feeding to include HAuCl_4_ under stirring and with magnetic separation to end the process. After washing with distilled water, NPs were protected with citrate ligands and stored in 10 mM sodium citrate buffer, pH 5.0. The obtained model nanocarriers were spherical particles 27.1 ± 3.0 nm in diameter. They contained a core of Fe_2_Ni 7.1 nm in diameter and a Au shell of 10 nm in thickness. The Au coating prevented corrosion of the superparamagnetic Fe_2_Ni core and provided plasmonic properties for the enhancement of Raman spectroscopic signals. The stability of nanocarrier dispersion depends on properties of the organic overcoat, forming the second shell on top of the Au shell, and was typically characterized by zeta potentials of −20 to −40 mV. The excess of negative charge on nanocarriers warranted colloid stability. Moreover, the excess of negative charge also blocked the random penetration of cell membranes, making the nanocarrier internalization totally dependent on the interactions of the targeting ligands attached to the nanocarrier with the respective receptor expressed on the target cancer cells. Spherical monodisperse gold nanoparticles were synthesized according to the procedure developed previously [69], based on citrate protective ligand and borohydride reducing agent. Reference gold nanoparticles were purchased from Nanopartz Inc. (Loveland, CO, USA).

All aqueous solutions were prepared using deionized water with 18.2 MΩ cm resistivity, purified with a Millipore purification system (Millipore, Inc., Bedford, MA, USA).

### 2.2. Modification of Au(111) Substrates with Nanocarrier Grids

**Scheme 1:**
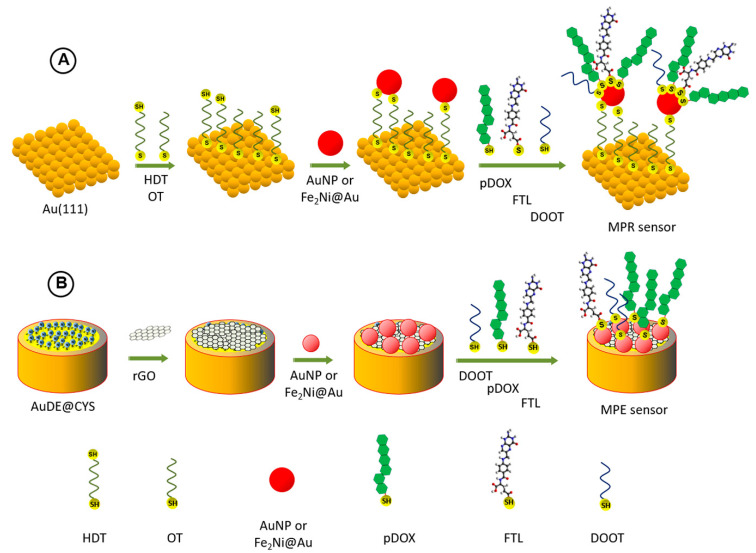
Construction of SERS-electrochemical sensors for the development of targeted drug nanocarriers: (**A**) magneto-plasmonic Raman (MPR) sensor based on a thin Au(111) basal film, with controlled-distance plasmonic NPs; (**B**) magneto-plasmonic electrode (MPE) for electrochemical transduction based on a gold disk electrode with immobilized plasmonic NPs. HDT—hexanedithiol; OT—octanethiol; pDOX—protected thiolated anticancer drug doxorubicin; FTL—thiolated folate targeting ligand; DOOT—biocompatibility agent 3,6-dioxa-octanethiol; rGO—reduced graphene oxide.

The sensor substrates made of a Au(111) film were cleaned by etching in freshly made piranha solution (3:1 *v*/*v* concentrated H_2_SO_4_: 30% H_2_O_2_; *CAUTION: Piranha solution is very dangerous, corrosive, and may explode if contained in a closed vessel; it should be handled with special care*). After 10 min etching, the substrates were thoroughly rinsed with Milli-Q water. To form a self-assembled monolayer (SAM) of a dithiol linker, a cleaned substrate was immersed in absolute ethanol solution containing 1.0 mM 1,6-hexanedithiol (HDT) and 10 mM 1-octanethiol (OT) (as diluent during the self-assembly process, to prevent the formation of bridged binding of HDT), with a volume ratio of 1:1, for 20 h. After the self-assembly process, Au sensors were thoroughly rinsed with ethanol and water to remove physically adsorbed HDT and OT, and then immersed in a solution of gold nanoparticles (GNPs) or magnetic nanoparticles (MNPs) for 3.5 h to form a monolayer grid of plasmonic NPs. The obtained nanoparticle grid was suitable for SERS measurements, as well as for electrochemical characterization. The obtained nanoparticle grid-modified Au(111) sensors were washed with deionized water and used for further functionalization with protected anticancer drug (pDOX), folate targeting ligand (FTL), and biocompatibility agent (DOOT). The steps leading to the immobilization of GNPs and MNPs on Au substrates and nanoparticles’ functionalization are illustrated in Scheme 1.

### 2.3. Doxorubicin Loading onto GNP and MNP Nanocarriers

The anticancer drug doxorubicin has a free NH_2_ group; therefore, it can be readily attached to the nanocarriers via linkers with carboxyl groups. For Au-coated MNPs and GNPs, the use of mercapto-propionic acid (MPA) as the linker was the appropriate choice. It forms a well-defined SAM on Au surfaces with strong thiolate bonding. Thus, the nanogrid electrodes, Au(111)@HDT/MNP and Au(111)@HDT/GNP, were soaked in 10 mM MPA solution for 4 h to form a linker SAM, followed by thorough washing with distilled water. The surface carboxyl groups of MPA were then activated by EDC/NHS coupling in DMSO medium for 1 h, and subsequently, a 0.5 mg/mL DOX solution in DMSO was added to complete the drug binding to the functionalized nanocarrier. After incubating for 4 h, the electrode was rinsed with DMSO and 3 times with water and saline phosphate buffer, pH 7.4. 

### 2.4. Simultaneous Functionalization of Nanocarriers with Anticancer Drug (DOX), Targeting Ligand (FA), and Biocompatibility Agent (DOOT)

**Scheme 2:**
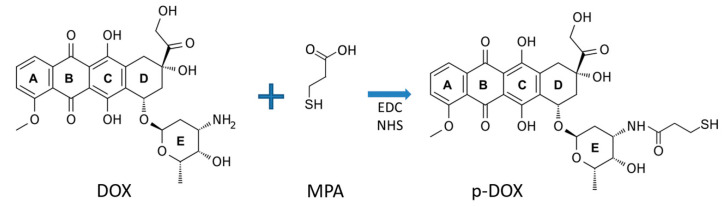
Binding of MPA linker to doxorubicin via the EDC/NHS-mediated amide bond formation process to protect the active NH_2_ group of the drug and provide a sulfhydryl moiety needed for attaching the drug to Au-coated MNPs.

Simultaneous functionalization of free nanocarriers, as well as the nanocarriers bound to nanogrid probes, with anticancer drugs, targeting ligands, and biocompatibility agents, was carried out using pre-thiolated compounds. Thus, doxorubicin was first coupled with MPA via EDC/NHS amide bond formation, as shown in Scheme 2. 

**Scheme 3:**
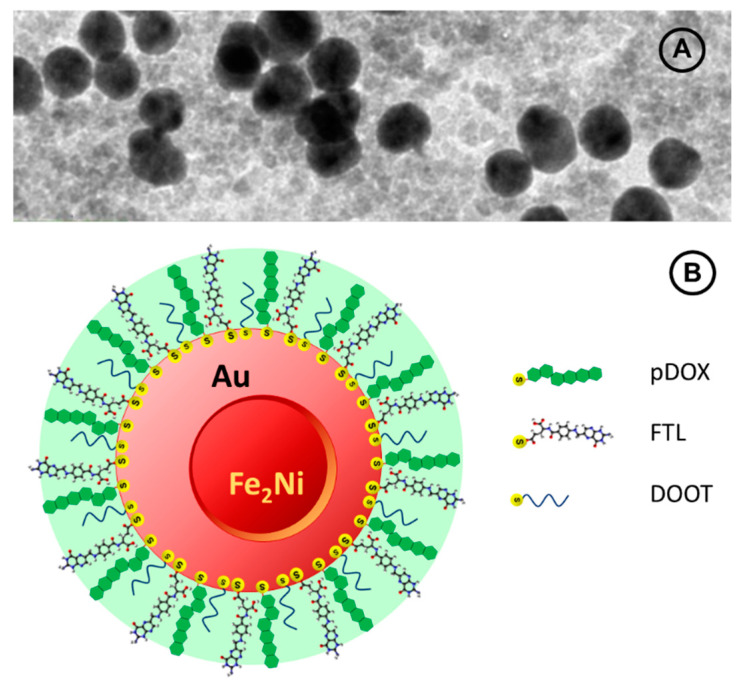
Magneto-plasmonic nanocarriers with Fe_2_Ni core and Au shell, coated with folate derivative as the folate targeting ligand (FTL), ethylene glycol derivative (DOOT) as the biocompatibility agent, and protected doxorubicin (p-DOX) as the chemotherapeutic drug: (**A**) TEM image of bare 27.1 ± 3.0 nm Fe_2_Ni@Au nanoparticles; (**B**) schematic view of the nanovector structure.

Folic acid targeting ligand, as the second component of the functionalizing solution, was obtained by activating FA with EDC/NHS, followed by bonding to cysteamine (CYS). The biocompatibility agent, 3,6,-dioxa-octanethiol (DOOT), in concentration 20 µg/mL, was used as the third component in the functionalization solution. It acts similarly to pegylated ligands used in implanted devices to protect them against opsonization and activation of immune responses. The nanogrid probes were dipped into the functionalization solution with component ratio: pDOX:FTL:DOOT = 20:1:1 and incubated for 1 h. The probes were thoroughly rinsed with PBS buffer saline, pH 7.4. A model MNP-based drug nanocarrier functionalized with pDOX, FTL and DOOT is depicted in Scheme 3.

### 2.5. Polycrystalline Gold Disk Electrode Modification for Electrochemical Detection

The modification of polycrystalline solid disk electrodes (AuDEs) for the loading of doxorubicin was similar to that published previously for investigations of the damage to DNA caused by chemotherapeutics [17]. Briefly, the gold disk electrode was cleaned, after polishing with alumina powder (0.05 µm in diameter), by scanning in a potential window between −0.4 and 1.7 V vs. SCE in 0.5 M H_2_SO_4_ at scan rate v = 100 mV/s, until the cyclic voltammograms for a clean Au electrode were obtained. The electrode was then modified with cysteamine HCl (CYS) as a linker for the attachment of graphene oxide (GO) nanosheets. The Au electrode was immersed in a 5 mM CYS solution for 30 min. After washing with distilled water, the AuDE@CYS electrode was immersed in an aqueous solution of GO with added EDC/NHS coupling agents for 4 h to attach single-layer GO sheets to the CYS linker by amide bonding with carboxyl groups of GO. The obtained AuDE@CYS/GO electrode was then washed in PBS buffer and distilled water. Next, the AuDE@CYS/GO electrode was activated with EDC/NHS coupling agents for 4 h. The CYS-capped MNPs (or GNPs) were then added to bind these nanoparticles to the AuDE/CYS/GO electrode surface. After incubation for 1 h, the electrode was rinsed with water and PBS buffer, pH 7.4. The obtained AuDE@CYS/GO/MNPs (or AuDE@CYS/GO/AuNPs) electrode was then scanned in 0.5 M NaCl solution from 0.7 to −1.1 V at a scan rate of 50 mV/s to reduce the GO and form a reduced graphene oxide (rGO) film [70]. Thus, the AuDE/CYS/rGO/MNPs and AuDE/CYS/rGO/AuNPs electrodes were obtained. They were then loaded with an anticancer drug or mixture of ligands to complete the immobilized-nanocarrier functionalization, as described in the sections above. 

### 2.6. pH-Responsive Release of Doxorubicin from Nanocarriers

The stimulated drug release from the surface of bound MNPs in Au(111)@HDT/MNPs@MPA/DOX sensors was monitored in solutions of different pH: in PBS at pH 7.4 and in acetate buffer solutions at pH 6.5, 5.3, and 4.5, using SERS measurements after different release times. The doxorubicin release was also investigated using voltammetric methods, CV and DPV, with a AuDE@CYS/rGO/MNP@MPA/DOX electrode.

## 3. Results and Discussion

In this work, magnetic Fe_2_Ni@Au nanoparticles (MNPs) were synthesized and functionalized with an anticancer drug, doxorubicin (DOX), and folic acid (FA) as the targeting ligand. A biocompatibility agent, 3,6-dioxa-octanethiol (DOOT), was also attached to MNPs to prevent the organism’s immunoresponse. The drug loading onto the nanocarriers was closely monitored with surface-enhanced RAMAN scattering (SERS) and electrochemical relaxation methods: cyclic voltammetry (CV) and differential-pulse voltammetry (DPV). The designs of the SERS sensor, formed on a Au(111) substrate, and the electrochemical sensor, formed on a polycrystalline Au-disk electrode (AuDE), are presented in Scheme 1. These two types of sensors had the following compositions:

(1) The SERS sensor, formed on a Au(111) substrate, was coated with a monolayer of magneto-plasmonic nanocarriers, bound to the substrate via a dithiolate linkage (HDT), as follows: Au(111)@HDT/MNP@MPA, where the solution side of the MNPs was coated with MPA after MNP binding to the substrate. The SERS sensor in this work refers to a magneto-plasmonic nanogrid Raman sensor (MPR sensor);
(2) The electrochemical sensor, formed on a polycrystalline Au-disk electrode (AuDE), was coated with two structural layers of rGO and MNP grid, and had the composition: AuDE@CYS/rGO@PATP/MNP@MPA. The use of the rGO basal layer enabled binding of MNPs via PATP linkage and blocking the diffusion of redox probe ions (Fe^2+^/Fe^3+^) to the AuDE substrate. The electrochemical sensor in this work refers to a magneto-plasmonic nanogrid rGO disk electrode sensor (MPE sensor).

These two types of sensor grids have enabled not only the monitoring of MNP nanocarrier functionalization, but also following of the stimulated drug release transients using solutions simulating the conditions of healthy and cancer cells. Thus, the proposed sensing platform can be utilized as a tool for the development of new drug nanocarriers, and serve in this role under simulated bio-environment conditions, outside of the real cells and tissues. By immobilizing new drug nanocarriers on the sensor surface, the NC characteristics can be monitored, including the content of drugs bound in the nanocarrier shell, the drug upload and release conditions, and nanodrug stability in different media. The attached nanocarriers can be investigated to optimize the nanocarrier design and content without expensive testing in a biological environment with cells and tissues. However, the sensing platform developed in this work was not designed to directly release nanovectors into the cells.

### 3.1. Assembling MPR Biosensors with Drug Nanocarrier-Mimetic NP Grids

**Figure 1:**
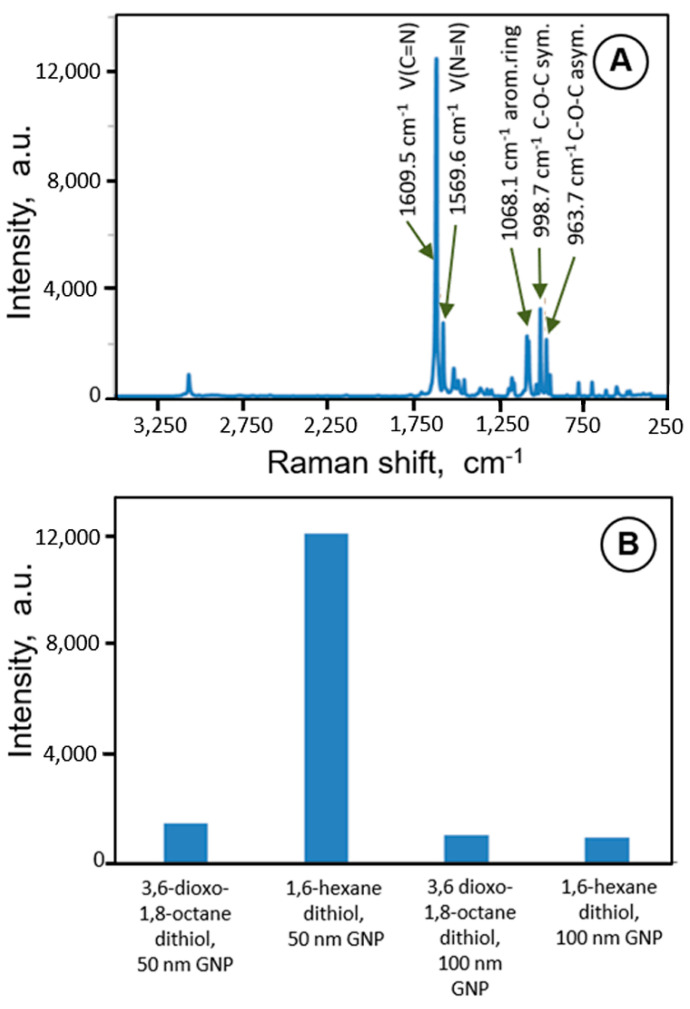
Testing the effects of different dithiol linkers and GNP sizes, using a Raman marker 5-phenyl-1,3,4-oxadiazole-2-thiol (PODAT), to optimize the GNP grid-enhanced SERS sensor for drug nanocarrier development. (**A**) SERS spectrum of PODAT adsorbed on a Au(111)/GNP-grid electrode surface; the Au(111)/GNP-grid electrode was obtained by depositing an SAM of 1,6-hexanedithiol (HDT) on a Au(111) substrate, followed by binding GNPs (50 nm in diameter) and adsorbing PODAT on the free surface of GNPs. (**B**) The effect of different dithiols and GNP size on the PODAT peak intensity at 1609 cm^−1^: (1) 3,6-dioxa-1,8-octanedithiol (DOODT) and 50 nm GNPs; (2) HDT and 50 nm GNPs; (3) DOODT and 100 nm GNPs; (4) HDT and 100 nm GNPs.

Raman sensors with high SERS amplification for studies of drug nanocarriers’ functionalization and anticancer drug loading and their release were designed on the basis of Au(111) substrates modified with a grid of NPs serving as a monolayer collection of drug nanocarrier-mimetic nanoparticles. Initially, to optimize the SERS sensor design, Raman markers were applied. In Figure 1A, an SERS spectrum of a Raman marker, 5-phenyl-1,3,4-oxadiazole-2-thiol (PODAT), adsorbed on a grid of GNPs bound to a Au(111) substrate, is presented. The Raman bands at 1609.5 cm^−1^ and 1569 cm^−1^ are assigned to vibrations of C=N and N=N groups of the adsorbed PODAT molecules, respectively. This evidence indicates that the Raman marker was successfully attached to the sensor surface using a dithiol linker and GNPs. The intensity of the scattering peak at 1609.5 cm^−1^ was then used for comparison with GNP grids with different NP sizes and for different dithiols used for GNP binding, as illustrated in Figure 1B for GNPs of 50 and 100 nm diameter and two kinds of dithiols: 3,6-dioxa-1,8-octanedithiol and 1,6-hexanedithiol. The recorded PODAT signal intensity depended on the surface area of GNPs available for Raman marker adsorption and on the plasmonic field enhancement due to the overlap of electromagnetic fields in narrow spaces between GNPs. As seen, the highest SERS intensity was obtained for 50 nm GNPs and the shorter linker, 1,6-hexanedithiol. Therefore, these conditions were utilized in further SERS sensor designs in this study. Similar size-dependent effects were found by Toro et al. [71] in studies of AuNP drug nanocarriers internalized in pancreas cancer cells. 

### 3.2. SERS Monitoring of Doxorubicin Loading onto Magnetic NP Nanocarriers

**Figure 2:**
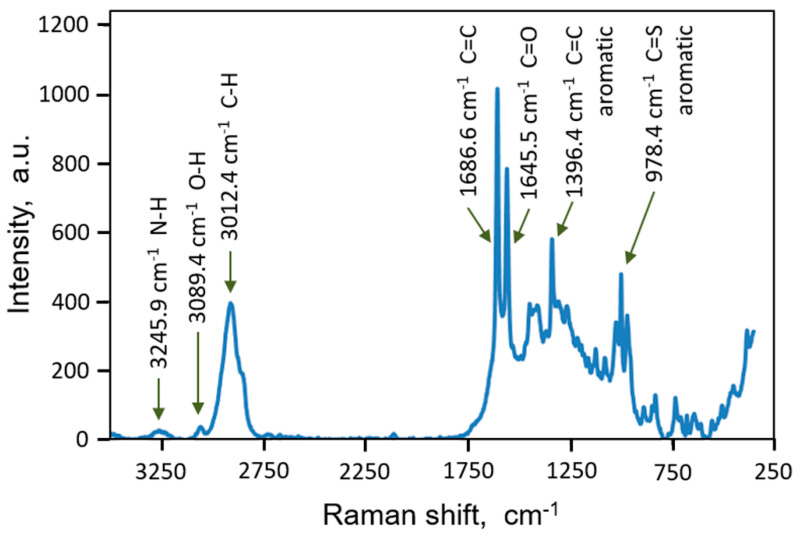
Raman spectrum for a protected anticancer drug doxorubicin (pDOX) bound to model GNP nanocarriers immobilized on an SERS biosensor, Au(111)@HDT/GNP_50nm_@pDOX.

The progress of loading of a protected DOX (pDOX) onto a GNPs-modified SERS sensor was monitored by recording the Raman spectra associated with loaded pDOX. A typical spectrum, presented in Figure 2, revealed several characteristic peaks at 1686 cm^−1^, 1645 cm^−1^, 1396 cm^−1^, 978.5 cm^−1^, 1396 cm^−1^, 3012 cm^−1^, 3089 cm^−1^ and 3246 cm^−1^. These Raman peaks are related to C=C, C=O, aromatic C=C, aromatic C=S, C–H, OH and N–H vibrations, respectively. The resulting spectra are in excellent agreement with data in the literature [72,73,74] and the DOX structure (Scheme 2). This also means that the substrate was covered successfully with pDOX. The appearance of peaks at 1645 and 3246 cm^−1^, corresponding to bending vibrations of CO and NH, indicated that doxorubicin was conjugated to GNP via an amide linkage to MPA (Figure 2).

### 3.3. Electrochemical Monitoring of Sensor Functionalization

Electrochemical analysis is very sensitive and can be readily employed in sensing applications [69]; therefore, we have also carried out measurements using stationary and relaxation voltammetric techniques, such as cyclic voltammetry (CV) and differential pulse voltammetry (DPV), for the characterization of MNP-modified gold probes. The use of electrochemical techniques enables monitoring and confirmation of nanocarrier modifications [27], as well as drug loading and release. In these measurements, we have applied a redox probe Fe^2+^/Fe^3+^ able to penetrate surface films and provide the information about changes in film permeation due to the surface modification, drug loading, and its release.

**Figure 3:**
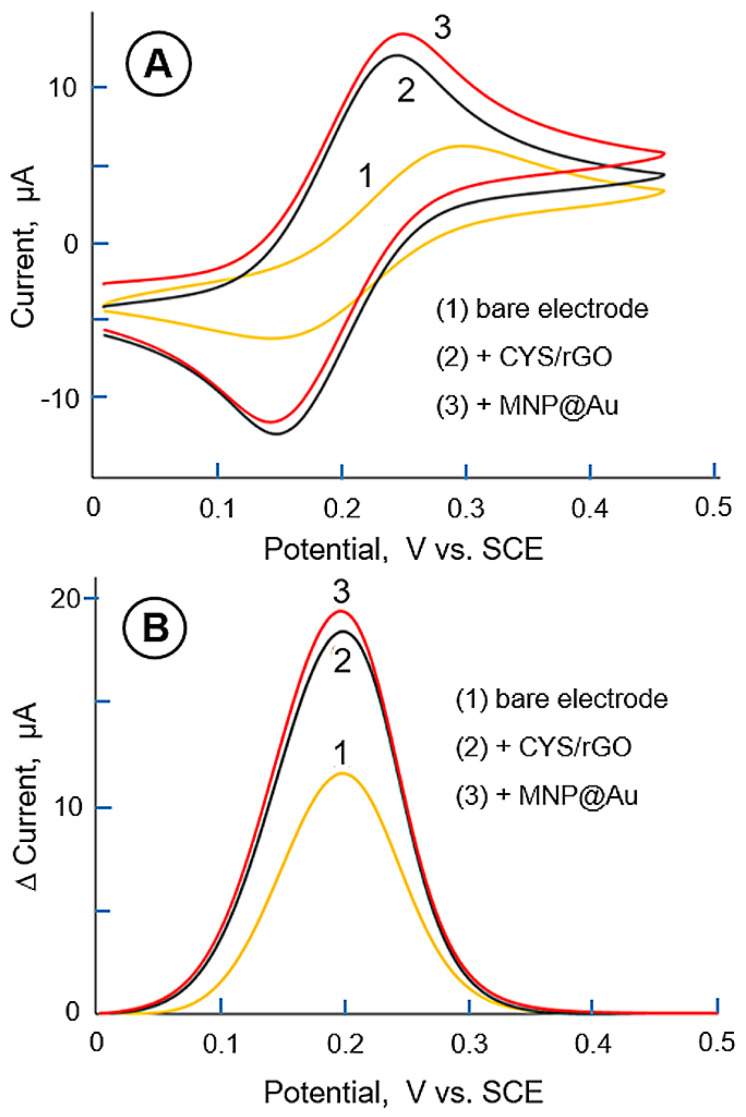
(**A**) Cyclic voltammograms for Fe^2+^/Fe^3+^ couple recorded using: (1) bare AuDE electrode, (2) AuDE/CYS/rGO, (3) AuDE/CYS/rGO/Fe_2_Ni@Au MPE sensor; scan rate *v* = 100 mV/s. (**B**) Differential pulse voltammograms for Fe^2+^/Fe^3+^ on: (1) bare AuDE electrode, (2) AuDE/CYS/rGO, (3) AuDE/CYS/rGO/Fe_2_Ni@Au MPE sensor; conditions: amplitude 0.05 V, pulse width 0.05 V; MNP 50 nm in diameter.

In Figure 3A, CV characteristics for a Raman probe undergoing modifications are presented. As can be seen, a pair of peaks between 0.1 V and 0.3 V is clearly discernible in all CVs. These peaks are attributed to the redox processes of an Fe^2+^/Fe^3+^ couple. The redox peak currents increase from the original height for a bare electrode (curve 1), to that for a modified electrode (curve 2), upon the electrode functionalization with a reduced graphene oxide film, AuDE/CYS/rGO. Further increase in peak currents is observed after binding GNPs on top of rGO (curve 3). 

Similar experiments were also performed using a differential pulse technique (Figure 3B). The same trend of the peak current increase upon functionalization of the probe with rGO and GNPs was observed. However, due to the better discrimination against capacitive current, the analytical signal of DPV could be determined with higher precision.

These experiments confirm the suitability of the electrochemical techniques for monitoring of the Raman probes’ functionalization, including the deposition of MNP and GNP grids and graphene oxide films.

**Figure 4:**
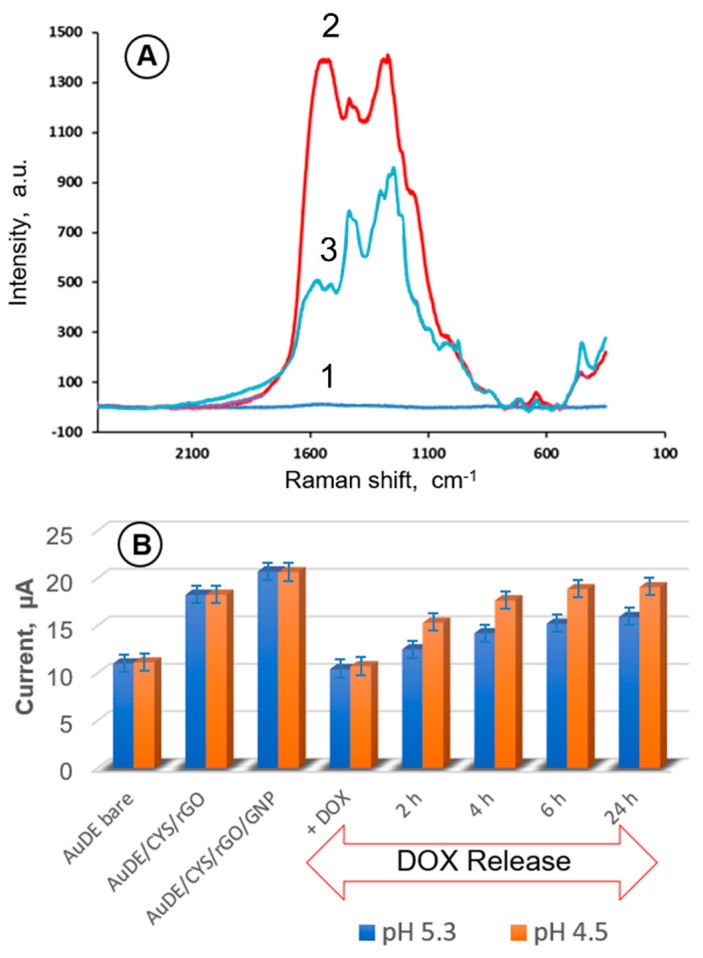
(**A**) Raman spectra for a protected anticancer drug doxorubicin (pDOX) adsorbed on model magneto-plasmonic nanocarriers (Fe_2_Ni@Au, 50 nm in diameter), immobilized on an MPR sensor: (1) blank sensor without any modification; (2) after incubation with pDOX; (3) after 4 h of drug release (pH 5.3). (**B**) DPV peak currents for the redox probe Fe^2+^/Fe^3+^ at a AuDE@CYS/rGO/MNP/pDOX electrode (MPE sensor) during the DOX release time, up to 24 h, at pH 5.3 (blue columns) and 4.5 (red columns).

In a similar way, electrochemical relaxation techniques were also tested for the monitoring of drug loading onto the nanocarriers. Representative DPV data are shown in Figure 4B, in the first four groups of blocks. These experiments indicate that the DPV oxidation current of the Fe^2+^ redox probe decreased upon DOX immobilization on the electrode surface, as expected, due to the increased blocking of the electrode surface, hindering the charge-transfer process, and slowing down the diffusion of redox probe ions through the NP film. 

### 3.4. DOX Encapsulation in Nanocarrier Shell

The encapsulation depends on the size of nanovectors; therefore, it is rational to compare the drug carrying capabilities and properties of nanocarriers for large and small nanovectors. Hence, we considered the following two representative types of nanocarriers: (i) large 50 nm nanocarriers, which provide optimized SERS amplification when attached to the magneto-plasmonic grid biosensing platform; and (ii) small nanocarriers, 3 nm in diameter which offer other clinical advantages, including passive tumor penetration and efficient clearance options through the kidney. 

Theoretical drug carrying capability of nanocarriers for DOX molecules can be calculated on the basis of DOX dimensions evaluated from quantum mechanical calculations of its electronic structure: *a* = 1.448 nm, *b* = 0.705 nm, and *c* = 0.925 nm, where *a* is the length, *b* is the thickness, and *c* is the width of the molecule (as drawn in Scheme 2). 

Thus, for a representative 27 nm nanocarrier with surface area *S*_NC_ = 2291 nm^2^, the maximum surface coverage by DOX in vertical orientation, with the base surface area *S*_DOX_ = *bc* = 0.6521 nm^2^, is *n*_DOX_ = 3513 molec/NC. This encapsulation ratio corresponds to the volumetric ratio of DOX to base vector *V*_DOX_/*V*_NC_ = 32.2%, and mass ratio of DOX to base vector *m*_DOX_/*m*_NC_ = 1.72%. These numbers represent saturated DOX coverage on nanocarriers. They will be lower when a mixed monolayer of DOX with FTL and DOOT is considered. Hence, for a DOX surface coverage of 80% and DOX molecules immobilized on the surface of nanocarriers in a vertical orientation (to protect the active functional groups of DOX against decomposition during transportation to the cancer cells, as discussed earlier), the number of DOX molecules carried by single nanocarriers is 2811. This corresponds to ca. 25.7% of the volume of a bare nanocarrier and 1.37% of the mass of a drug-loaded nanocarrier. The nanocarriers with this large drug loading can reduce the exposure to highly toxic chemotherapeutic drug to a minimum level, because the free DOX concentration typical in conventional whole-body administration of 630 ng/mL (*C*_max_ in blood plasma during IV infusion [75]), or 1.09 µM DOX, would be replaced with 386 pM concentration of inactive nanocarriers carrying protected DOX molecules. At the same time, the local dosage at the target cancer cells will be increased to enhance the treatment efficacy.

In case of a small, 3 nm nanocarrier with surface area *S*_NC_ = 28.3 nm^2^, the maximum surface coverage by DOX in vertical orientation is *n*_DOX_ = 43.4 molec/NC, which corresponds to a volumetric ratio of DOX to base vector *V*_DOX_/*V*_MNP_ = 0.05%, and mass ratio of DOX to base vector *m*_DOX_/*m*_MNP_ = 0.005%. These numbers represent saturated DOX coverage on nanocarriers, and they will be lower if 20% of the surface coverage is needed for the targeting ligand and biocompatibility agent. However, a single nanocarrier will still be able to carry large number of DOX molecules: *n*_DOX_ = 34.7 molec/NC.

### 3.5. Monitoring of pH-Induced Drug Release with SERS-Electrochemical Sensors

**Figure 5:**
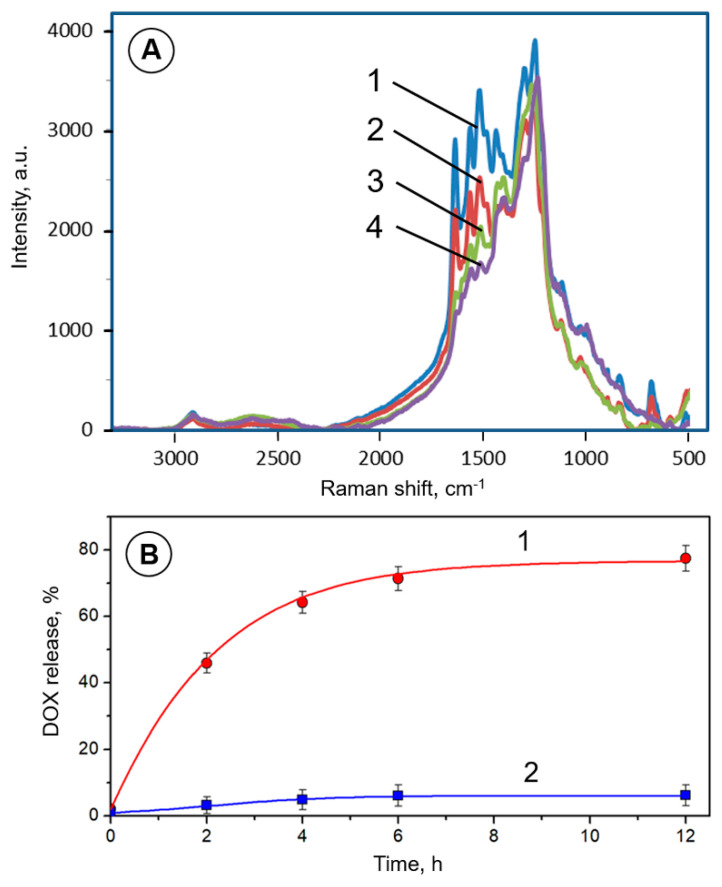
(**A**) Raman spectra of an MPR sensor with a monolayer of MNPs (Fe_2_Ni@Au) modified with protected doxorubicin (pDOX), thiolated folate ligand (FTL), and biocompatibility ligand DOOT, recorded after drug release times *t*_rel_ [h]: (1) 0 h; (2) 2 h; (3) 4 h; (4) 6 h, under simulated cancer cell conditions with pH = 5.3. (**B**) Temporal evolution of DOX release from MNP nanocarriers, attached to the MPR sensor, determined from changes of the intensity of Raman scattering peak of DOX at 1651 cm^−1^ for: (1) pH 4.5, and (2) pH 7.4.

Testing of magneto-plasmonic nanocarrier grid sensors, MPR and MPE, was performed for sensors functionalized with the protected anticancer drug pDOX (Figure 4), as well as for multifunctional sensors with model nanocarriers coated with pDOX, folate targeting ligand (FTL), and biocompatibility ligand DOOT (Figure 5).

The investigations of DOX release have been carried out in solutions with acidities below the physiological pH 7.4, to simulate the conditions of cancer cells. In tumor tissues, a pH value of 6 or less is readily attained, because during the generation of ATP by the anaerobic glucose metabolism, with lactic acid formation as a byproduct, a large number of hydrogen ions are produced. Even lower pH values, in the range 3.0–5.5, are encountered within the cancer cells in acidic intracellular organelles, such as the endosomes and lysosomes [24]. Hence, in the simulation of cancer cell conditions, the drug release experiments were performed at pH 5.3 and 4.5 (Figure 4). The CVs and DPVs were obtained for 2, 4, 6 and 24 h of DOX release time.

The drug loading and release from model nanocarriers attached to the sensor surface were monitored using SERS spectroscopy. In Figure 4A, SERS spectra are presented for: a bare MPR sensor (curve (1)), a sensor after uploading DOX onto the model nanocarriers (curve (2)), and a sensor after 4 h of drug release at pH = 5.3. The pronounced differences between the Raman scattering intensities observed under these conditions demonstrate the high sensitivity of the SERS probe for drug loading and release. In Figure 4B, the anodic peak currents observed in DPV experiments upon the amide bond acidolysis and DOX release process are shown. The obtained results indicate that the drug release efficacy is the highest during the first 2 h of the interaction between the model MNP@pDOX nanocarriers immobilized on an MPE sensor and solutions, with faster release observed for pH 4.5, as expected. During the first 2 h, 46.2 ± 2.3% of DOX was released, and around 65.4 ± 2.5% of the drug was released during the first 4 h. After 24 h, the DOX release amounted to 84.0 ± 3.1%.

The release of drugs from pH-responsive nanocarriers is due here to the acidolysis of amide bonds. The mechanism of this process was thoroughly studied by Szostak et al. [76] and was recently discussed by Running et al. [42] for amide-bonded dabrafenib utilized in a nanocarrier-based drug delivery system for melanoma treatment. The effects of electron-withdrawing properties of acid and amine substituents in asymmetric amides and amide bond twisting were indicated as amide bond weakening factors of this otherwise stable binding. Thus, amides may hydrolyze in aqueous acidic solutions (pH ≤ 6), releasing the amine fragment (DOX) to the solution [77]. 

### 3.6. Doxorubicin Delivery Using Pegylated Nanocarriers with Targeting Folate Ligands

To achieve targeted delivery, the surfaces of MNPs were further modified with folic acid (FA). The MNPs functionalized with thiolated folate ligands (FTL) in their shells can recognize folate-receptors overexpressed in the membranes of cancer cells. Binding of MNPs by FA-receptors was followed by endocytosis and induced drug release in cytosol. The ligands improving nanocarrier’s biocompatibility were also attached to the MNP surface. We found it convenient to utilize 3,6-dioxa-octanethiol (DOOT) for this purpose. The drug release from MNP@(pDOX, FTL, DOOT) nanocarriers was investigated using SERS in solutions simulating pH conditions of cancer cells. The SERS spectra, presented in Figure 5A, show a decrease in Raman scattering intensity during the course of DOX release, up to 5 h, in a solution of pH = 5.3. 

The DOX release kinetic transients are presented in Figure 5B. As can be seen from curve (1), the drug release is not linear over time. At pH 4.5, ca. 64.2 ± 2.5% of loaded DOX was released from MNP nanocarriers during the first 4 h, and 77.5 ± 3.1% of loaded DOX was released in a 12 h period. These results indicate that the DOX release from model nanocarriers functionalized with FTL and DOOT is lower than that in the absence of FTL and DOOT, but the decrease is only within a ca. 6.5% range. 

The results obtained for DOX loading onto the model nanocarriers and its release under simulated intracellular conditions corroborate the utility of the magneto-plasmonic nanoparticle grid platform developed in this work as a convenient and highly sensitive SERS-electrochemical biosensing device for designing and characterizing novel nanocarriers for targeted drug delivery. The proposed methodology exploits the principles of nanodrug designs [1,2,9,12,13,14,17,41], including drug protection from the biological medium, mitigation of collateral damage to healthy cells, selective targeting of cancer cells based on biorecognition interactions between nanocarrier-bound ligands and complementary receptors expressed on cancer cells, and the prevention of opsonization and immune system attack.

## 4. Conclusions

A new nanoparticle grid-based biosensing platform for developing drug nanocarriers for targeted delivery systems has been designed and tested. The proposed sensor grid, consisting of a monolayer of model magnetic gold-coated core–shell Fe_2_Ni@Au nanoparticles, was functionalized with folic acid targeting ligand, model protected chemotherapeutic drug doxorubicin (pDOX), and a biocompatibility agent, 3,6-dioxa-octanethiol (DOOT). The dual signal transduction employed in the proposed sensor, based on electrochemical and enhanced Raman scattering detection, enabled convenient monitoring of DOX loading onto the nanocarriers and its release under simulated intracellular conditions. We have demonstrated a pH-dependent release of DOX from drug nanocarriers immobilized on the sensor surface, under intracellular low-pH conditions of cancer cells. The enhanced sensitivity of Raman scattering was achieved owing to the overlap of the plasmonic fields emanating from Au-shells of the model drug nanocarriers, creating “hot-spots” for Raman signals’ amplification. The proposed nanocarrier grid devices can serve for designing and optimizing novel drug nanocarriers for new, safe TDD systems, which will mitigate adverse side effects and high collateral damage to non-tumorigenic cells caused by systemic drug administration. The main purpose of this work was to design and test a new device and methodology for developing new nanocarriers for targeted drug delivery. Therefore, this work was focused on the construction of model nanocarrier arrays able to create plasmonic “hot spots” that enabled the highly sensitive detection of Raman signals associated with drug loading onto the nanocarriers, attachment of targeting ligands, and methods of protection of nanocarriers against opsonization and attacks by the patient’s own immune system. The proposed device also enabled monitoring of the drug release process under simulated intracellular conditions of the cancer cells. These basic investigations can then be followed by in vitro and in vivo studies of the performance and efficacy of the developed nanocarriers by medical personnel in healthcare facilities. Due to the expanding applications of targeted drug nanocarriers’ technology, magneto-plasmonic biosensing platforms have a high potential to become developmental tools for designing new nanovectors for fighting cancer and other diseases.

## Data Availability

Data are contained within the article.

## Abbreviations

AuDE	gold disk electrode
CV	cyclic voltammetry
CYS	cysteamine
DOOT	biocompatibility agent (3,6,-dioxa-octanethiol)
DOX	doxorubicin
DPV	differential pulse voltammetry
EDC	carboxyl activating agent (1-Ethyl-3-(3-dimethylaminopropyl) carbodiimide hydrochloride)
FA	folic acid
FR	folate receptor
FTL	thiolated folate targeting ligand
GNP	gold nanoparticles
GO	graphene oxide
HDT	hexane dithiol
MNP	magnetic nanoparticles
MPA	mercaptopropionic acid
MPE	magneto-plasmonic electrode
MPNP	magneto-plasmonic nanoparticle
MPR	magneto-plasmonic Raman probe nanocarrier
NC	nanocarrier
NHS	N-hydroxysuccinimide, (CH_2_-CO)_2_NOH
OT	octanethiol
PATP	4-aminothiophenol
pDOX	protected DOX
PEG	polyethylene glycol
PODAT	5-phenyl-1,3,4-oxadiazole-2-thiol
rGO	reduced graphene oxide
SAM	self-assembled monolayer
